# The effect of enhancing quality of life in patients intervention for advanced lung cancer: Protocol for a randomized clinical study

**DOI:** 10.1097/MD.0000000000023682

**Published:** 2020-12-18

**Authors:** Xianhong Li, Ke Qin, Chunyan Yuan, Shiqiang Song

**Affiliations:** a Department of Urology; b Department of Rehabilitation Medicine, People's Hospital of Chengyang District, Qingdao, China.

**Keywords:** enhancing quality of life in patients, lung cancer, protocol

## Abstract

**Objective:**

The objective of this present research is to evaluate the effect of the intervention of enhancing quality of life in patients in patients with advanced lung cancer.

**Methods:**

Our research is carried out as a randomized clinical trial which will be implemented from December 2020 to October 2021. It was approved by the Ethics Committee of People's Hospital of Chengyang District (03982790). This study includes 90 patients with advanced lung cancer. Patients diagnosed at our oncology clinic are eligible if they are diagnosed within 8 weeks of a novel diagnosis of stage 3 or stage 4 lung cancer. Patients with hepatic insufficiency, renal failure, and respiratory and heart failure, as well as a series of severe mental illness are excluded from our research. Patients are divided randomly into the intervention group and control group, each group is assigned 45 patients. Through utilizing functional assessment of cancer therapy–lung, the measurement of life quality is conducted. And the measurement of mood is carried out with Hospital Anxiety and Depression Scale.

**Results:**

Table 1 indicates the patient's life quality and Hospital Anxiety and Depression Scale in both groups.

**Conclusion:**

Enhancing quality of life in patient intervention may be beneficial to improve the life quality in advanced lung cancer patients.

Trial registration: The protocol was registered in Research Registry (researchregistry6243)

## 1 Introduction

Lung cancer is the main cause of cancer in the world; approximately 1.6 million patients are diagnosed with lung cancer every year.^[1,2]^ It is the most familiar cause of deaths associated with cancer, with 1.3 million people dying from the disease each year, accounting for 1/5 of cancer deaths. Most lung cancers can be divided into small-cell lung cancer or non-small cell lung cancer. At the time of diagnosis, most patients have metastatic cancer or locally advanced cancer that is incurable, with a 5-year mortality rate of 85% to 90%.^[3]^ The treatment method for lung cancer is determined by the type of lung cancer, the disease stage, and the performance status of patients.^[4]^ The treatment methods involve radiation therapy, chemotherapy, surgical resection, palliative care as well as targeted therapy.^[5,6]^

The goal of treatment is to improve the life quality and prolong the life of patients with advanced lung cancer, or both. Nevertheless, due to poor functional status, many patients have limited treatment options. Many factors may lead to low functional status, involving comorbidities, high tumor burden, and older age.^[7]^ For the cancer progression, its direct effects include weight loss, shortness of breath, pain, and fatigue, together with some indirect influences of the cancer treatment, can accumulate to further deterioration in the life quality, sustained loss of physical health, and a decline in levels of physical activity.^[8,9]^ In patients with advanced lung cancer, the palliative care may have the greatest benefit, which may be due to the low life quality and heavy burden of symptoms of advanced lung cancer patients.^[10]^

The intervention of enhancing quality of life in patients (EQUIP) is a kind of supportive nursing intervention for the advanced lung cancer patients, which is dominated by nurses. It consisted of four educational sessions, led by a research nurse, on how to deal with the prevalent lung cancer symptoms, that is, worry, breathlessness, and fatigue. The objective of this present research is to evaluate the effect of the intervention of EQUIP in the advanced lung cancer patients.

## 2 Methods

### 2.1 Study design

This experiment is implemented as the randomized clinical study which will be conducted from December 2020 to October 2021. After obtained approval from the Ethics Committee of People's Hospital of Chengyang District (03982790), we registered it in research registry (researchregistry6243). This study includes 90 patients with advanced lung cancer. With the use of the random number table, all patients will be assigned the random number, and the distribution results will be hidden in the random envelope. All the patients are randomly assigned to the control and intervention group, each group is assigned 45 patients.

### 2.2 Inclusion of exclusion criteria

Patients diagnosed at our oncology clinic are eligible if they are diagnosed within 8 weeks of a novel diagnosis of stage 3 or stage 4 lung cancer. Patients with hepatic insufficiency, renal failure, and respiratory and heart failure, as well as a series of severe mental illness are excluded from our research.

### 2.3 Intervention in both groups

In the control group and the intervention group, the patients are given routine care, that is, access to all palliative and oncology treatment services. Furthermore, the EQUIP intervention group undergoes four face-to-face meetings with the nurse. All courses are carried out by the same research nurse with the experience of community palliative care and the registered nurse qualification. The research nurse only participates in the implementation of EQUIP intervention; the evaluations of patient reports are managed via a separate study coordinator who is unaware of the allocation of two groups. The EQUIP courses arrangement is consistent with the existing outpatient chemotherapy or clinic visits time, so as to minimize the inconvenience to the patients. The focus of these courses is to equip the patients with the strategies and skills to deal with pervasive symptoms of lung cancer patients. This content includes the following aspects:

(1) Fatigue: introducing the patients to the nonpharmacological treatment of fatigue, containing maintaining activity vitality and rhythm;
(2) Breathlessness: introducing nonpharmacological treatment of breathlessness to the patients, involving relaxation techniques and breathing, fan therapy, and mindfulness;
(3) Worry: introducing the psychobehavioral strategies to the patients to deal with worry; and
(4) Recap of the former sessions. After ending each session, the patients are given a 1-page summary of content.

### 2.4 Data collection

With the functional assessment of cancer therapy–lung, the measurement of life quality can be conducted.^[11]^ The 36-item tool measures emotional, physical, social, emotional, functional health and the symptoms specific to lung cancer. With Hospital Anxiety and Depression Scale, the measurement of mood is carried out.^[12]^ This 14-item tool consists of 2 subscales for depression and anxiety. This is a kind of categorical scale, and cut-off scores for each subscale are as follows: 0 to 7 represents normal, 8 to 10 represents mild, 11 to 14 represents moderate, and 15 to 21 represents severe.

### 2.5 Statistical analysis

All the data can be recorded into the software of Microsoft Excel 2013, and the analysis of all the data is carried out through utilizing the software of IBM SPSS Statistics for Windows, version 20 (IBM Corp., Armonk, NY). All the data are represented via the proper characteristics, for instance, median, mean, and percentage. The categorical variables and continuous variables are respectively analyzed using independent *t*-tests and χ^2^-tests. *P* value less than .05 indicates that there is statistical significance.

## 3 Results

Table 1 indicates the patient's life quality and Hospital Anxiety and Depression Scale in both groups.

**Table 1:** Patient's life quality and HADS in both groups.

Outcomes	Study group (n = 45)	Control group (n = 45)	*P* value
Physical well-being			
Social well-being			
Emotional well-being			
Functional well-being			
Overall quality of life			
Anxiety subscale			
Normal			
Mild			
Moderate			
Severe			
Depression subscale			
Normal			
Mild			
Moderate			
Severe			

## 4 Discussion

Lung cancer is one of the prevalent malignant tumors in the world.^[13,14]^ Like all cancers, the best hope for a cure for lung cancer is to early detect. Nevertheless, many lung cancer patients are diagnosed as advanced and often cannot be cured.

Palliative care is a concept in health care that has been developed over the past decade to solve the needs of supportive care associated with life-threatening diseases.^[15]^ Early palliative care can improve the symptom burden, patients’ quality of life, and enhance the survival rate.^[16]^ With the extensive evidence supporting the effect of palliative care in the improvement of patient outcomes, a new challenge is to optimize the resources to conduct effective services. EQUIP intervention is jointly developed via the interdisciplinary team of healthcare professionals, and these healthcare professionals have the relevant clinical expertise in locally managing lung cancer patients, involving professional palliative care nurses and care physicians, physiotherapists, and clinical psychologists. This is the first research to confirm the acceptability and feasibility of EQUIP intervention in advanced lung cancer patients. Further high-quality trial is still required to confirm our results.

## 5 Conclusion

EQUIP intervention may be beneficial to improve the life quality in advanced lung cancer patients.

## Author contributions

**Conceptualization:** Ke Qin.

**Data curation:** Ke Qin.

**Funding acquisition:** Shiqiang Song.

**Investigation:** Chunyan Yuan.

**Methodology:** Chunyan Yuan.

**Writing – original draft:** Xianhong Li.
